# Achieving Intraperitoneal Disease Control Using Cytoreductive Surgery and Hyperthermic Intraperitoneal Chemotherapy: Two Cases of Metastatic Breast Cancer

**DOI:** 10.7759/cureus.38767

**Published:** 2023-05-09

**Authors:** Philipp Barakat, Vadim Gushchin, Luis Felipe Falla Zuniga, Mary Caitlin King, Armando Sardi

**Affiliations:** 1 Surgical Oncology, Mercy Medical Center, Baltimore, USA

**Keywords:** peritoneal spread, peritoneal neoplasms, peritoneal metastases, hipec, breast neoplasms

## Abstract

Peritoneal metastases from breast cancer (PMBC) tend to occur late in the disease course and are challenging to manage. Cytoreductive surgery and hyperthermic intraperitoneal chemotherapy (CRS/HIPEC) provide peritoneal disease control in other malignancies and may achieve similar results in PMBC. We assessed intraperitoneal disease control and outcomes in two PMBC patients after CRS/HIPEC. Patient 1, diagnosed at age 64, had hormone-positive/human epidermal growth factor receptor 2 (HER2)-negative lobular carcinoma treated with mastectomy. Prior to salvage CRS/HIPEC at age 72, five cycles of intraperitoneal chemotherapy via an indwelling catheter failed to control recurrent peritoneal disease. Patient 2, diagnosed at age 52, had hormone-positive/HER2-negative ductal-lobular carcinoma and received lumpectomy, hormonal therapy, and target therapy. Prior to salvage CRS/HIPEC at age 59, she had recurring ascites that was resistant to hormonal therapy and required multiple paracenteses. Both underwent complete CRS/HIPEC with melphalan. The only major complication was anemia, which required a transfusion in both patients. They were discharged on postoperative days 8 and 13, respectively. Patient 1 had peritoneal recurrence 26 months post-CRS/HIPEC and died of disease at 49 months. Patient 2 never had peritoneal recurrence and died of extraperitoneal progression at 38 months. In conclusion, CRS/HIPEC is safe and can provide intraperitoneal disease and symptom control in select patients with PMBC. Thus, CRS/HIPEC can be offered to these rare patients who have failed standard treatments.

## Introduction

With many therapeutic advancements in recent decades, metastatic breast cancer is usually well managed with hormonal therapy, chemotherapy, radiation, and supportive care in the majority of patients. However, there may be a subgroup of patients with isolated metastatic disease that can benefit from surgery to achieve disease control [1-3].

Peritoneal metastases (PM) always pose a clinical and therapeutic challenge given the insidious onset of symptoms, unique anatomic biology, and complicated drug delivery [4,5]. PM from breast cancer are rare, often occur late in the disease course after failing multiple treatments, and have poor response to chemo/hormonal therapy, which limits the number of available therapeutic options for these patients [6-8]. Symptoms are often recurring, significantly affecting quality of life, and treatment guidelines for such cases have not been established yet.

Cytoreductive surgery and hyperthermic intraperitoneal chemotherapy (CRS/HIPEC) provide good peritoneal disease control in patients with a variety of other malignancies that commonly present with extensive abdominal spread. Experienced centers report excellent patient safety outcomes [9]. We hypothesized that similar results can be achieved in patients with PM from breast cancer who have failed other therapies. We assessed intraperitoneal disease control and outcomes in two cases of PM from breast cancer treated with CRS/HIPEC.

This was previously presented as an abstract and poster at the Third Annual ISSPP (International Society for the Study of Pleura and Peritoneum) Congress on October 13, 2022, in Huntington Beach, California.

## Case presentation

Patient information

Patient 1

Patient 1 had a medical history significant for osteoporosis and gastric ulcer. She also had a family and personal history of breast cancer with negative BReast CAncer gene (BRCA) status. She had progesterone receptor (PR)-positive, estrogen receptor (ER)/human epidermal growth factor receptor 2 (HER2)-negative ductal carcinoma of the right breast with 0/15 lymph nodes positive treated with lumpectomy and radiation at age 49, as well as ER/PR-positive, HER2-negative lobular carcinoma of the right breast measuring 1.2 cm with no lymphatic invasion (T1cNx) treated with mastectomy at age 64. At age 66, she was diagnosed with stage IIIa, T1cN2M0 ER/PR-positive, HER2-negative ductal carcinoma of the left breast treated with mastectomy, adjuvant chemotherapy, radiation, and hormonal therapy (letrozole and tamoxifen). Four years later, a polyp biopsy on a routine colonoscopy showed metastatic lobular breast carcinoma. Subsequently, she underwent hemicolectomy with transverse colectomy. Final pathology was consistent with ER/PR-positive, HER2-negative metastatic adenocarcinoma of mammary origin with 4/33 lymph nodes involved.

Though asymptomatic, follow-up PET/CT five months post-hemicolectomy showed omental and peritoneal thickening consistent with peritoneal disease. She received five cycles of intraperitoneal (IP) cisplatin every three weeks that was stopped due to intolerance, which impacted her quality of life. IP chemotherapy failed to achieve intra-abdominal disease control, with rising tumor markers and positive peritoneal washings six months after therapy.

Patient 2

Patient 2 had a significant medical history of depression, gastroesophageal reflux disease (GERD), and chronic pancreatitis. She also had a family history of cancer in both parents, but she was never tested for BRCA mutations. At age 52, she was diagnosed with pT1N0M0 ER-positive, PR/HER2-negative ductal-lobular carcinoma of the right breast but initially refused treatment. Six months later, she underwent a right lumpectomy/sentinel lymph node biopsy, which was negative. Further treatment was declined, except for radiation.

At age 58, she presented with abdominal discomfort, bloating, and weight gain. The work-up did not show evidence of disease until a CT scan the following year demonstrated ascites with cytology consistent with metastatic adenocarcinoma of the breast primary. She started anastrozole but progressed with increasing ascites after 1.5 months. She underwent four paracenteses and multiple trials of letrozole and palbociclib with stable disease after seven months of treatment. Follow-up PET and CT scans showed ascites and peritoneal carcinomatosis without extraperitoneal disease. The patient was dissatisfied with the quality of life and effect of treatment.

CRS/HIPEC treatment

The decision to proceed with CRS/HIPEC was based on the failure of previous treatment to adequately control intraperitoneal disease and case-specific factors. Patient 1 had a short-term response to IP chemotherapy with cisplatin, suggesting a potential need for a more radical approach. Patient 2 desired a surgical approach after little relief from medical management. Both patients were presented at a multidisciplinary cancer conference and educated about the availability of other treatments, potential complications, and paucity of data regarding the efficacy of CRS/HIPEC in breast cancer.

The age at CRS/HIPEC was 72 years for patient 1 and 59 years for patient 2. The number of major resections was five and seven for patients 1 and 2, respectively. Abdominal disease burden, quantified intraoperatively using the peritoneal cancer index (PCI) (range: 0-39), was 14 in patient 1 and 29 in patient 2. The completeness of cytoreduction score (CC score) was 1 (residual scar tissue <0.25 mm) in both cases. HIPEC was performed with melphalan (50 mg/m^2^) at a temperature of 41-43°C using the closed technique for 90 minutes. Both patients had postoperative anemia requiring a transfusion. No other major morbidity occurred. Both spent one day in the intensive care unit (ICU) and were discharged on postoperative days 8 and 13, respectively, after they were able to eat, no longer required IV pain medication, and were able to walk without assistance, per our standard practice. Neither patient required enteral feeding, and abdominal drains were removed once output decreased to <150 mL/day. More information is shown in Table 1.

**Table 1:** Perioperative characteristics for each patient. *Right lumpectomy with radiation; right mastectomy with no adjuvant treatment; left mastectomy with radiation and chemotherapy with docetaxel/cyclophosphamide; letrozole for 10 months; tamoxifen for 2.5 years; hemicolectomy for colon metastasis followed by exemestane; fulvestrant for four months; intraperitoneal cisplatin for five cycles. **Right lumpectomy with radiation; anastrozole for two months; letrozole/palbociclib for six months and restarted after CRS/HIPEC. ***Splenic flexure, previous ileocolonic anastomosis, omentectomy including the gastroepiploic arcade, resection of liver capsule segments 2-6, right and left diaphragmatic peritonectomy. ****Bilateral parietal peritonectomy, right diaphragmatic peritonectomy, resection of liver capsule segments 1 and 4-8, portal dissection, omentectomy, splenectomy, hysterectomy, bilateral salpingo-oophorectomy. Overall and progression-free survival are calculated from the date of CRS/HIPEC to date of death and date of initial recurrence, respectively. AJCC: American Joint Committee on Cancer; BRCA: BReast CAncer gene; CC score: completeness of cytoreduction score; CRS/HIPEC: cytoreductive surgery and hyperthermic intraperitoneal chemotherapy; CTCAE: Common Terminology Criteria for Adverse Events; DOD: dead of disease; ER: estrogen receptor; G: grade; HER2: human epidermal growth factor receptor 2; ICU: intensive care unit; LN: lymph node; PCI: peritoneal cancer index; PR: progesterone receptor.

	Patient 1	Patient 2
Age at diagnosis, years	64	52
Age at CRS/HIPEC, years	72	59
Stage	T1cNx (AJCC 7)	T1N0M0, Ia (AJCC7)
Pre-CRS/HIPEC histology	Lobular carcinoma, ER/PR positive, HER2 negative	Ductal-lobular carcinoma, ER positive, PR/HER2 negative
Post-CRS/HIPEC histology	Lobular carcinoma, ER positive, PR/HER2 negative, 0/11 LN positive	Ductal-lobular carcinoma, ER/PR positive, HER2 negative, 0/13 LN positive
BRCA status	Negative	Unknown
Treatment before CRS/HIPEC	Multiple*	Multiple**
Prior surgical score	1	0
PCI at exploration	14	29
PCI post-CRS/HIPEC	4	3
CC score	CC-1	CC-1
Site of residual disease	Small bowel mesentery (scarring)	Small bowel mesentery (scarring)
Length of surgery, minutes	424	540
Number of major resections	5***	7****
HIPEC agent	Melphalan	Melphalan
Length of ICU stay, days	1	1
Length of stay, days	8	13
Complications (CTCAE v.5)	Anemia (G3), thrombocytopenia (G1)	Anemia (G3), thrombocytopenia (G2), oral thrush (G2)
Postoperative therapy	Raloxifene	Letrozole + palbociclib
Initial site of recurrence	Rising tumor markers	Liver parenchyma
Progression-free survival, months	5	2
Time to peritoneal recurrence, months	26	None
Status	DOD	DOD
Overall survival, months	49	38

Follow-up and outcomes

Follow-up included a physical exam, CT scans, and tumor markers every three months. The patients were also closely followed by their medical oncologists.

After surgery, patient 1 received raloxifene. Initial disease recurrence, diagnosed by rising tumor markers with no visible lesions, occurred five months after surgery. Due to positive androgen receptors, she was switched to enzalutamide. After multiple lines of treatment, peritoneal recurrence occurred 26 months after CRS/HIPEC. Patient 1 succumbed to the disease 49 months post-CRS/HIPEC.

Patient 2 received postoperative therapy with letrozole and palbociclib. Two months post-CRS/HIPEC, liver lesions were noted, but since tumor markers were declining, it was decided to continue observation. A subsequent CT scan five months post-CRS/HIPEC confirmed the enlargement and metastatic origin of the liver lesions. Patient 2 never had peritoneal recurrence but died of disease 38 months post-CRS/HIPEC.

## Discussion

The peritoneum is associated with poor drug delivery and concentration through intravenous access [4]. In addition, the immunologic interaction between the omentum and peritoneum results in anti-inflammatory activities and can be altered by cancer, leading to a suppressed immune response [5]. This can hinder treatment success, which, in combination with debilitating symptoms and poor quality of life, creates an unmet need for an effective approach to control PM.

Despite a wide range of conservative breast cancer therapies, their efficacy in the setting of PM remains low. CRS has proven to be an effective way to address peritoneal involvement in other cancers. After removal of all visible disease, HIPEC assists with intraperitoneal disease control via direct drug delivery to the abdomen, increased concentration in the tumor and peritoneum, and enhanced cytotoxic effects from hyperthermia [10]. Thus, in combination, CRS/HIPEC can help overcome the obstacles unique to peritoneal biology and potentially improve outcomes. Van Driel et al.’s randomized phase III trial showed improved overall and progression-free survival with the addition of HIPEC to CRS compared to CRS alone in patients with ovarian cancer after neoadjuvant chemotherapy [11]. Pseudomyxoma peritonei (PMP), commonly presenting with voluminous, mucinous ascites, has also been successfully managed with CRS/HIPEC, the current standard of care that provides excellent survival outcomes [12,13]. Given its proven success in other malignancies with significant peritoneal spread, CRS/HIPEC may be an option to control peritoneal disease in select cases of breast cancer with PM.

Information about the efficacy of CRS/HIPEC in breast cancer patients is largely limited to retrospective case series. In the biggest multicenter study, Cardi et al. reported 49 patients with PM from ductal and lobular breast cancer with variable molecular subtypes divided into two treatment groups: patients having CRS with or without HIPEC for curative intent versus those who had noncurative interventions including drug therapy, palliative surgery, and intraperitoneal chemotherapy. There was improved overall survival in the CRS with/without HIPEC group (89.2% vs. 6% alive at 36 months, p < 0.001) [1]. However, this study included patients with incomplete CRS (CC-2, residual disease 2.5 mm-2.5 cm), the presence of preoperative extraperitoneal metastases in the CRS group, and the utilization of HIPEC based on each center’s policy. Another study by Cardi et al. reported five cases of breast cancer treated with CRS/HIPEC with disease-free survival ranging from 13 to 128 months [2]. Yu et al. reported four cases with overall survival ranging from 15 to 49 months [3]. Both studies included patients with isolated PM from ductal and lobular cancers with different molecular status. Our patients had isolated PM from lobular and ductal-lobular carcinomas with an overall survival of 49 and 38 months, respectively. Achieving complete cytoreduction (CC-1, only minimal residual scarring) may explain the good peritoneal disease control in our patients, with only one of the patients recurring to the peritoneum over two years post-CRS/HIPEC. Thus, CRS/HIPEC seems to provide peritoneal disease control in PM from breast cancer.

In the reported data, response to CRS/HIPEC in breast cancer is variable, suggesting a need for more evidence to determine the effect of stage, prior treatment, and histology. However, it is clear that isolated peritoneal disease can benefit from locoregional therapy and should be confirmed when assessing the feasibility of CRS/HIPEC [1-3,10-13]. We aimed to attain peritoneal disease control that was not achieved with prior interventions without jeopardizing patient safety. A multidisciplinary conference agreed that CRS/HIPEC would be a reasonable alternative treatment approach for these symptomatic patients, despite the high likelihood of systemic recurrence.

When selecting a treatment option, particularly for terminal, metastatic cancers, maintaining patient safety and quality of life is essential. Unfortunately, we cannot expect large-scale or randomized trials in the near future, and the available studies, including ours, suffer from a small sample size. However, based on our experience in the management of peritoneal disease, CRS can be performed safely at experienced centers. Interestingly, the addition of HIPEC to CRS does not affect the rates of adverse events [11]. Our results support this statement with the only major morbidity being anemia, requiring a transfusion in both patients.

Long-term quality of life in CRS/HIPEC patients has been a subject of debate. Several studies have demonstrated that the majority of patients return to baseline within one year of CRS/HIPEC [14,15]. The addition of HIPEC to CRS alone has also been shown to be safe [16,17]. Our experience is in line with these data. Thus, when considering the risks and advantages of CRS/HIPEC in this setting, we based our judgment on the assumption that the initial detriment to quality of life postoperatively will be outweighed by abdominal symptom control and expected survival benefit greater than one year. Despite an initially poor prognosis related to peritoneal spread, after CRS/HIPEC, both patients had meaningful survival of more than two years and resolution of peritoneal disease, which had failed to be controlled by alternative therapies and was causing significant symptoms.

We understand the controversy surrounding a radical surgical approach in metastatic breast cancer and agree that CRS/HIPEC should be reserved for select patients. We believe that the clinical decision to proceed with CRS/HIPEC should be based on a history of failing multiple standard treatments to control peritoneal disease, the absence of visceral metastatic lesions, and the experience of the center. Each patient should be counseled on the potential risks of the approach, lack of prospective data, likelihood of recurrence, and alternative treatment options.

This study’s limitations are its retrospective nature, small number of patients, and different tumor histologies. These factors decrease our ability to make conclusions about overall and progression-free survival. However, having achieved localized disease control, we provide comprehensive data on rare cases successfully managed with an unconventional approach.

## Conclusions

In these two cases, CRS/HIPEC was safe and provided intraperitoneal disease and symptom control in breast cancer with PM. Clinicians may consider CRS/HIPEC in select patients who have failed traditional treatment modalities. More research is needed to further improve patient selection.
